# How can long-term cold storage affect the content of glomalin-related soil proteins from an arbuscular mycorrhizal inoculum?

**DOI:** 10.1007/s10482-026-02428-1

**Published:** 2026-09-20

**Authors:** Eduarda Lins Falcão, Fábio Sérgio Barbosa da Silva

**Affiliations:** 1 Laboratório de Análises, Pesquisas e Estudos em Micorrizas (LAPEM) and Programa de Pós-graduação em Biologia Celular e Molecular Aplicada, Centro de Pesquisas do Instituto de Ciências Biológicas, Universidade de Pernambuco (UPE), Rua Arnóbio Marques, 310, Santo Amaro, Recife, PE 50100-130 Brazil; 2 Universidade de Pernambuco, Av. Agamenon Magalhães, S/N, Santo Amaro, Recife, PE 50100-010 Brazil

**Keywords:** *Entrophospora*, *Glomeromycota*, Glycoproteins, Shelf-life

## Abstract

It has been shown that cold long-term storage of *Entrophospora etunicata* inoculum may decrease the concentration of spore proteins. However, no studies have been carried out to understand how long-term storage under cold conditions may interfere with glomalin-related soil proteins (GRSP) concentration, thermostable glycoproteins released by arbuscular mycorrhizal fungi (AMF). To elucidate how this shelf-life factor may modulate GRSP concentration, the same isolate of *E. etunicata* was cultivated in 2000 (*Ee2000*) and 2023 (*Ee2023*) and stored since then under cold conditions (4–6ºC). The easily extractable fraction of GRSP (EE-GRSP) was extracted from these inocula using 0.25g of soil-inoculum, equivalent weight for 50 spores in the soil-inoculum, and a spore suspension with 50 spores. The results were expressed as the concentration in µg for each approach evaluated and as the Carbon of GRSP (C-GRSP). In the treatment of equivalent weight for 50 spores, the EE-GRSP and C-GRSP concentrations were reduced by 35% in *Ee2000* in comparison to *Ee2023*, indicating that such a reduced concentration of GRSP and C-GRSP may occur in stored inocula at a slow rate due to cold storage. A slight decrease (9.5%) in EE-GRSP and C-GRSP was observed in *Ee*2000 soil-inoculum from 0.25g treatment, and no difference in protein concentration was shown in the spore suspension. It is concluded that *E. etunicata* inoculum has its EE-GRSP and C-GRSP negatively affected by long-term storage in cold conditions. This is the first study to report the behaviour of GRSP in cold long-term stored AMF inoculum.

**Graphic Abstract:**

**Figure:**
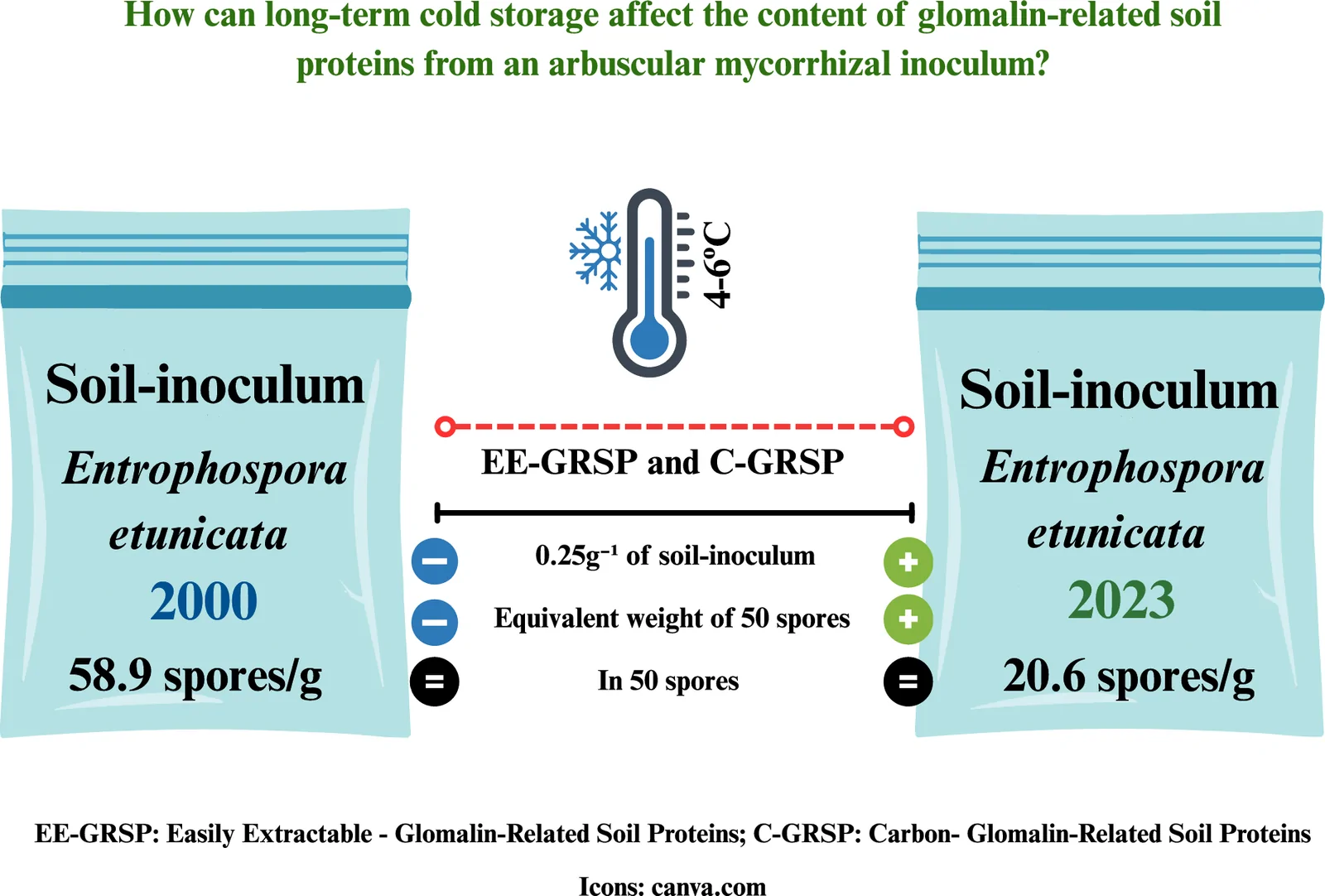

## Introduction

The effects of 24–26 years of *Entrophospora etunicata* (W.N. Becker & Gerd.) Błaszk., Niezgoda, B.T. Goto & Magurno (*Glomeromycota*) inoculant storage impairs its capacity for colonization, viability, and even interferes with total protein preservation in spores of this arbuscular mycorrhizal fungal (AMF) species (Falcão and Silva 2025; Falcão et al. 2026). In this context, a protein group that can persist for several years in soil is the glomalin-related soil proteins (GRSP) (Mou et al. 2025), recalcitrant glycoproteins synthesized in the hyphae and spores of these microorganisms (Purin and Rillig 2008). These molecules, which have N-type linkage to carbohydrates (Wright et al. 1998) and high chelating capacity (Son et al. 2024), are homologous to heat-shock protein 60 (Gadkar and Rillig 2006) with high thermal stability (up to 300 °C) and a potential source of iron for plants and microorganisms (Liu et al. 2026a).

The aforementioned characteristics underscore the extensive role of GRSP in the soil environment, ranging from improving soil aggregate stability (Ji et al. 2024), remediation (Zhou et al. 2024), regulation of the soil microbiome (Zhou et al. 2026), adsorption of phytotoxic metabolites (Liu et al. 2026b), and contributing to soil organic Carbon (Yang et al. 2024). Moreover, microorganisms may decompose the sugar portion of these molecules, influencing their concentration in soil (Rillig et al. 2003) regardless of their known long-term persistence in soils (Mou et al. 2025). Notwithstanding, the behaviour of these protein glycoconjugates in long-term stored inocula is not known.

In this regard, AMF inoculum naturally contains microorganisms, and some of them found in *E. etunicata* isolates may have a psychrophilic nature (Pandit et al. 2022). Notwithstanding, no studies have investigated how long cold inoculant storage can affect GRSP concentration in AMF inocula, despite it being known that GRSP decomposition occurs, especially from the easily extractable fraction (EE-GRSP), which is more sensitive to environmental shifts (Emran et al. 2026) and remarkably influences soil dynamics upon stress factors (Li et al. 2026).

Therefore, given that GRSP have primary relevance to AMF structures (Purin and Rillig 2007), but also can be decomposed by microbiota (Rillig et al. 2003), in this study it was investigated whether long-term storage under cold conditions may reduce the concentration of EE-GRSP and its Carbon (C-GRSP). This was achieved by testing the hypothesis that GRSP concentration from an *E. etunicata* soil-inoculum is reduced after 26 years of storage under cold conditions. The aim was to elucidate how long inoculant storage may affect the preservation of GRSP in an AMF inoculum stored for 26 years at 4–6°C.

## Materials and methods

To evaluate how 26-year storage (28th January 2000 to 20th April 2026) may affect the concentration of EE-GRSP and its Carbon, inocula from *E. etunicata* used in previous research (Falcão and Silva 2025; Falcão et al. 2025; 2026) were chosen for this study. The most recent and the oldest inoculum come from the same isolate provided by the Mycology Department (*Universidade Federal de Pernambuco*, Brazil). The isolate is registered on the *Sistema Nacional de Gestão do Patrimônio Genético e do Conhecimento Tradicional Associado* (SisGen) (ABC3019).

### Description of *Entrophospora etunicata* inoculum production

The first cultivation of *E. etunicata* occurred in 2000 (*Ee2000*), using a sterilized substrate composed of sand and vermiculite (1:1, v/v) (two cycles, 121°C, 15min, in two consecutive days) and *Panicum miliaceum* L. as host (50 seeds per pot of 400mL). The plants were watered with nutrient solution [KNO_3_ 1.5×10^−3^ mol L^−1^, Ca(NO_3_)_2_ 1.5×10^−3^ mol L^−1^, MgSO_4_ 0.3×10^−3^ mol L^−1^, KH_2_PO_4_ 3×10^−6^ mol L^−1^, FeNa EDTA 45×10^−6^ mol L^−1^, NaCl 30×10^−6^ mol L^−1^, ZnSO_4_ 0.7×10^−6^ mol L^−1^, CuSO_4_ 0.3×10^−6^ mol L^−1^, MnCl_2_ 9.5×10^−6^ mol L^−1^, Na_2_MoO_4_ 0.066×10^−6^ mol L^−1^, H_3_BO_3_ 46.2×10^−6^ mol L^−1^] supplemented with Tris–HCl Buffer 50mM (pH 6.5) thrice a week (Hoagland and Arnon 1950, modified by Jarstfer and Sylvia 1992, and Silva et al. 2005). These plants were cultivated in greenhouse conditions at environmental conditions of temperature (max: 32.4°C; min: 22.8°C) and humidity (max: 81.1%; min: 45.6%). At the flowering stage, which occurred after 85 days, watering was ceased, the substrate was completely dried, and the aerial part was removed. Subsequently, the roots were cut (1 cm), homogenized into the substrate, and stored in resealable plastic bags at 4–6°C. The spore concentration of 58.9 spores g^−1^ soil was assessed by wet sieving (Gerdemann and Nicolson 1963) and centrifugation in sucrose solution (Jenkins 1964). The infectivity of 3.72% and viability of 35% were evaluated using the mean infection percentage (INVAM 2026) and MTT (3-(4,5-Dimethylthiazol-2-yl)− 2,5-Diphenyltetrazolium Bromide) assay (An and Hendrix 1988; Walley and Germida 1995, adapted), respectively. Further details of the methodologies and data are not presented in this study, as they can be found in Falcão and Silva (2025).

The most recent *E. etunicata* was subcultivated in 2023 (*Ee*2023) using the same substrate composition, host, and watering regime. The plants were also kept in environmental conditions of temperature (max: 34.8ºC; min: 23.1ºC) and humidity (max: 97%; min: 56%). After 79 days, watering was ceased, and the same process of drying and storage was repeated. This inoculum has 20.6 spores g^−1^ soil, 31.8% infectivity, and 92% viability, which were assessed using the same methods carried out for *Ee*2000 inoculum (Falcão and Silva 2025). For more information concerning some biochemical parameters and the efficiency of these inocula, see Falcão et al. (2025;2026).

### Experimental design

A completely randomised experiment using *E. etunicata* soil-inoculum stored for two different terms (*Ee2000* and *Ee2023*) with three biological replicates was set up.

### Easily extractable glomalin-related soil protein extraction

**Fig. 1:**
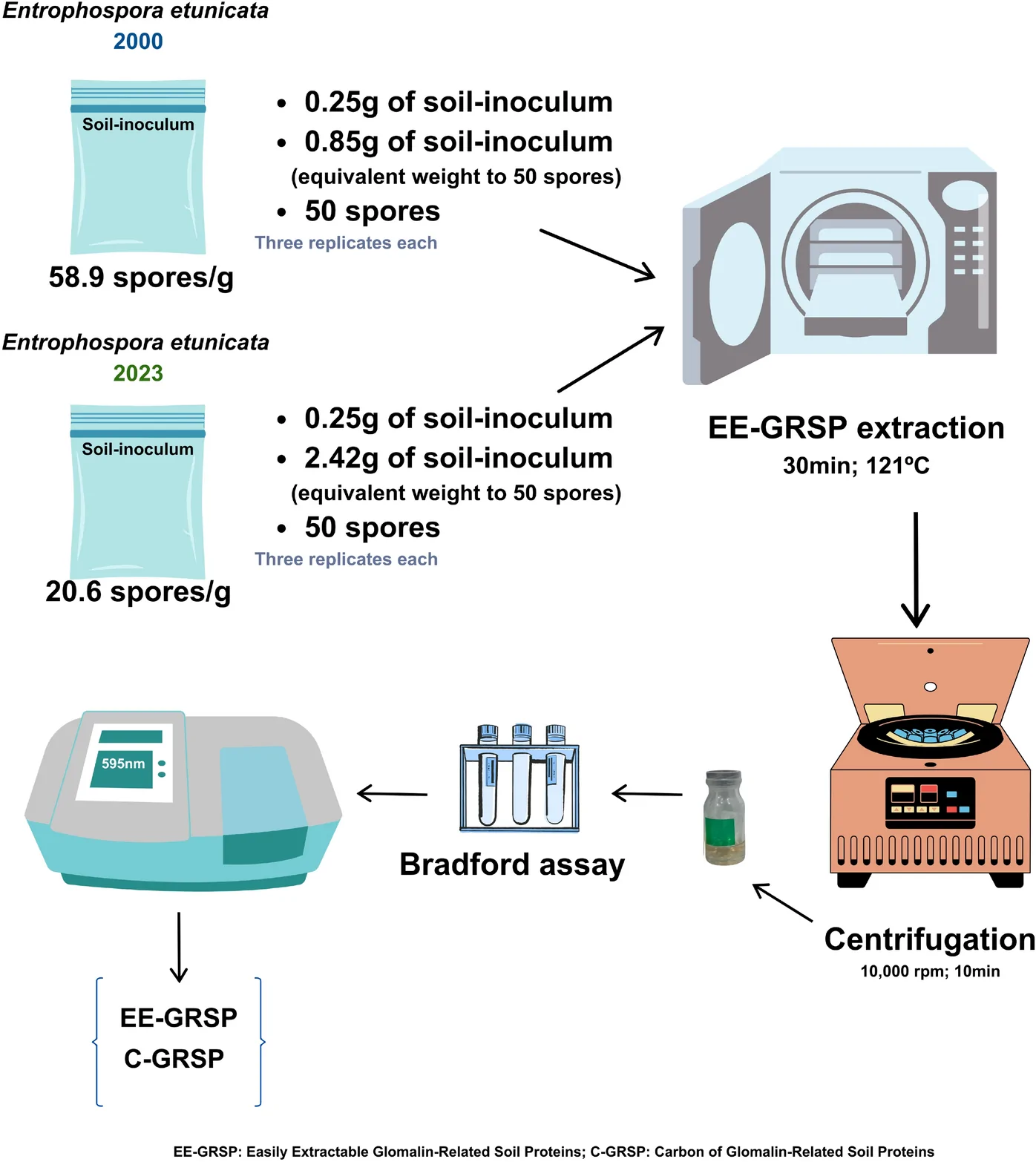
Step-by-step of the methodological procedures to evaluate the effect of long-term storage on *Entrophospora etunicata* (W.N. Becker & Gerd.) Błaszk., Niezgoda, B.T. Goto & Magurno inoculum on GRSP concentration and C-GRSP. Icons from canva.com

The EE-GRSP fraction was chosen in this study for being more sensitive to environmental shifts of these glycoproteins (Emran et al. 2026). To consider many extraction procedures as possible, the concentration of this fraction was evaluated considering three approaches (Fig. 1): 1) considering 0.25g plus 2mL of citrate buffer (20mM, pH 7) (Nuclear®, Diadema, São Paulo—Brazil), 2) extraction using equivalent weight of soil inoculum containing 50 spores (0.85g and 2.42g for *Ee2000* and *Ee2023*, respectively) and proportional volume of citrate buffer, 20mM, pH 7 (6.8mL and 19.36mL, respectively), and 3) 50 spores suspension by extraction using wet sieving followed by sucrose (Usina Ipojuca S.A., Ipojuca, Pernambuco—Brazil) centrifugation (Gerdemann and Nicolson 1963; Jenkins 1964). The EE-GRSP of these samples was extracted according to Wright and Upadhyaya (1998) by autoclaving the soil inoculum samples for 30min at 121°C (Stermax Produtos Médicos Ltda., Pinhais, Paraná—Brazil), and centrifugation for 10min at 10,000 × *g* (Stermax Produtos Médicos Ltda., Pinhais, Paraná—Brazil). The supernatant was recovered and stored (− 18°C) for protein quantification.

To evaluate the total EE-GRSP of *Ee2000* and *Ee2023* inocula, the Bradford assay was chosen for being used to quantify GRSP (Moragues-Saitua et al. 2019) and for being sensitive to low protein concentration (5µg/mL) (Bradford 1976). Thus, 0.05mL of EE-GRSP samples and 2.5mL of Bradford reagent (phosphoric acid—Dinâmica®, Indaiatuba, São Paulo—Brazil, Coomassie blue G-250—Vetec®, Duque de Caxias, Rio de Janeiro—Brazil, and ethanol—Neon®, Suzano, São Paulo—Brazil) were vortex-stirred. After 5min, the samples were spectrophotometrically read at a 595nm wavelength (Thermo Fisher Scientific®, Franklin, Massachusetts—United States). The bovine serum albumin (Sigma-Aldrich®, Barueri, São Paulo—Brazil) was used to generate the calibration curve (*y* = 0.0007*x* – 0.0063; *R*^2^ = 0.99). The results were expressed as µg of EE-GRSP in 0.25g, in equivalent weight of soil-inoculum containing 50 spores, and in 50 spores. These results were also expressed as C-glomalin, considering that these proteins may contain up to 43.1% Carbon (Rillig et al. 2003).

### Statistics

The data were normally distributed by Shapiro–Wilk test, and the means were compared by t-test (95%) (Assistat version 7.7). The graphs were plotted using the ChiPlot website (https://www.chiplot.online/) (Chiplot 2026).

## Results and discussion

**Fig. 2:**
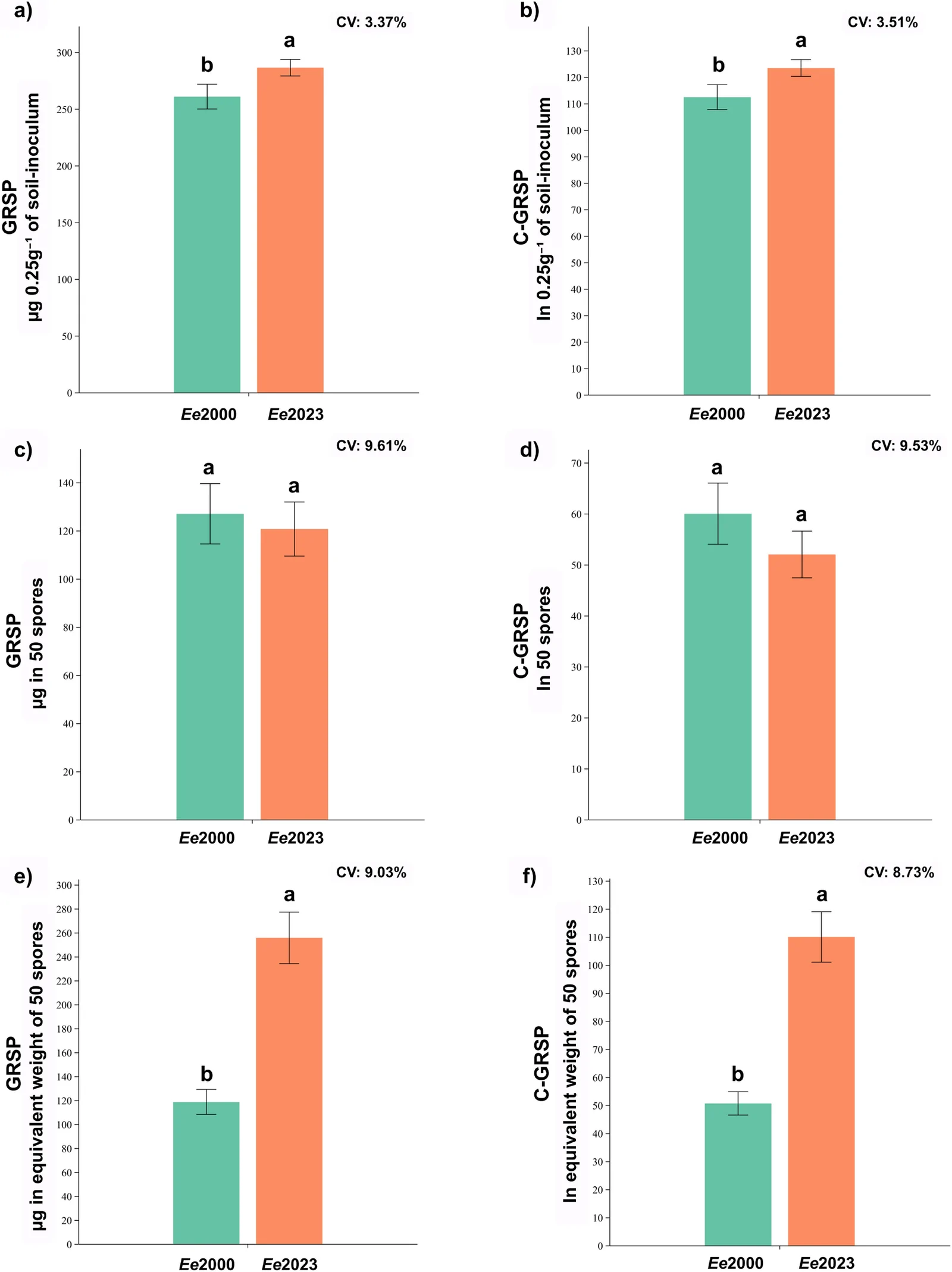
Values of glomalin-related soil proteins (GRSP, easily extractable fraction) and Carbon of GRSP (C-GRSP) from *Entrophospora etunicata* (W.N. Becker & Gerd.) Błaszk., Niezgoda, B.T. Goto & Magurno soil inoculum cultivated in 2000 (*Ee2000*) and 2023 (*Ee2023*) and stored at 4–6°C since then, considering A) GRSP in 0.25g of soil-inoculum, B) C-GRSP in 0.25g of soil-inoculum, C) GRSP in a suspension of 50 spores, D) C-GRSP in a suspension of 50 spores, E) GRSP in equivalent weight of a soil-inoculum containing 50 spores, F) C-GRSP in equivalent weight of a soil-inoculum containing 50 spores. Means (*n* = 3) followed by the same letter do not differ by the t-test (95%). Bars represent the standard deviation of the means. CV: Coefficient of Variation. The graphs were plotted using the ChiPlot website (https://www.chiplot.online/) (Chiplot 2026)

The EE-GRSP concentration ranged from 260µg to 290µg in 0.25g of soil inoculum, being slightly lower in *Ee2000* inoculum (Fig. 2). The same pattern was observed in the treatment using soil-inoculum with an equivalent weight of 50 spores, with a 35.6% increase in *Ee*2023 soil-inoculum (Fig. 2). On the other hand, long-term storage of *E. etunicata* inoculant does not seem to affect EE-GRSP extract from a suspension containing 50 glomerospores (Fig. 2).

Concerning the C-GRSP, regardless of the treatment, the same pattern was observed: *Ee2000* had its C-GRSP decreased when treatment from soil-inoculum was considered. The long-term storage did not affect C-GRSP from spore suspension (Fig. 2). Higher levels of C-GRSP potentially indicate that a greater preservation of Carbon sites in GRSP extracted from the soil inoculum from *Ee2023*. As seen by Schindler et al. (2007), some GRSP extracts can have more than 50% Carbon, which is divided into aliphatic, carbohydrate, aromatic, carboxyl, and carbonyl types that contribute to the backbone of these molecules.

Comparatively, the values of EE-GRSP found for *E. etunicata* inoculum obtained by Nichols (2010) in the third culturing period of a soilless culture using *Z. mays* as host were 0.36 ratio. On the other hand, Lovelock et al. (2004) reported 16µg mg^−1^ from the immunoreactive fraction of glomalin; nevertheless, it is worth noting that this study considered only the hyphal biomass and this research did not. For GRSP in the spore suspension, despite Alptekin et al. (2025) not being able to quantify GRSP from *Rhizoglomus irregulare* (Błaszk., Wubet, Renker & Buscot) Sieverd., G.A. Silva & Oehl spores using Bradford reagent, something not observed in the present study (Fig. 2). However, it is worth noting that the concentration of these glycoproteins may vary in AMF species (Hernandez 2001; Nichols 2010).

It is important to point out that the decrease in protein concentration of *Ee2000* spores previously reported is not likely related to these glycoproteins due to the extraction method (Falcão et al. 2026). Thus, it is known that a higher concentration of GRSP is found on the mycelium of AMF species than in spores; nevertheless, these propagules have a fast turnover (Staddon et al. 2003), which may explain the slight decrease of EE-GRSP concentration in *Ee2000* soil inoculum after 26 years. Despite being reported that, without the presence of a host, GRSP content can decrease by almost 50% (Rillig et al. 2003), this process may have been slowed down due to storage temperature. This result, once again, underscores the need for cold conditions to improve the shelf-life of AMF inoculants.

Chronosequence approaches have shown that GRSP may persist in soil for 42 years (Rillig et al. 2001) to up to two million years (Mou et al. 2025). In this matter, EE-GRSP may increase with the decrease of AMF biomass (Mou et al. 2025). Although this pattern was not observed in this study, it is important to highlight that these are projections with different types of soil, and in this case, a direct quantification in a soilless substrate was carried out (Fig. 2).

Despite being indicative that long-term storage may influence EE-GRSP concentration in AMF inocula, some limitations of this study need to be investigated in further research. Therefore, it is important to quantify the GRSP concentration of fresh AMF inoculum and repeat this process every year, followed by cold storage, so that at each point at which GRSP concentration starts to decay can be assessed.

The slight decrease in EE-GRSP and C-GRSP could be indicative of bacterial activity at a slow rate. As observed by Pandit et al. (2022), *E. etunicata* inocula may present *Enterobacter* strains, such as *Enterobacter ludwigii* 299,767, which can grow in cold conditions (Alikkunju et al. 2016). Notwithstanding, the carriers of these inocula were not the same as those used in this study, nor were the isolation sites.

Moreover, it would be important to determine the microbiome of these inoculants and whether it has changed over time. Does cold long-term storage favour a specific genus of psychrophilic bacteria that may decompose EE-GRSP? Does the carrier have a relevant role in this process? These are some of the questions that still need to be unravelled. To this end, it is important to have a great amount of long-term stored mycorrhizal inoculum to test the aforementioned methodologies periodically.

Another issue that should be considered is the Bradford assay itself. Despite being widely chosen in GRSP studies, many variables, such as the presence of phenolics, may interfere with the final protein concentration (Jorge-Araújo et al. 2015). Thus, it would be interesting to develop a specific monoclonal antibody for *E. etunicata*, since the only one available is more specific for *Rhizoglomus intraradices* (N.C. Schenck & G.S. Sm.) Sieverd., G.A. Silva & Oehl, which has been shown to also interact with carbohydrate sites from the AMF cell wall (Alptekin et al. 2025). Future studies should also consider proteomic approaches using long-term stored AMF inoculant, something understudied in AMF inoculum (Murphy et al. 2020), which may be important in understanding the physiology of *E. etunicata* long-term stored in cold conditions. These approaches may assist not only in the understanding of *E. etunicata* physiology, but also in the advancement of AMF inoculant storage research (Falcão and Silva 2026).

It is crucial to note that, to the best of our knowledge, no studies have been conducted to evaluate how long-term storage of AMF inoculum may affect the preservation of GRSP. The closest approach has been carried out by Selvakumar et al. (2018), which assessed the effect of long-term subculture (three years) on AMF isolates to promote GRSP deposition in *Capsicum annuum* L. rhizosphere soil.

The hypothesis of the study was partially confirmed, as EE-GRSP and C-GRSP of *E. etunicata* inoculum are affected by long-term cold storage, but this trend does not occur when only spores are considered. It is concluded that *E. etunicata* inoculum has its GRSP negatively affected by long-term storage in cold conditions, consequently reducing the Carbon content of these glycoproteins. These findings are relevant to AMF inoculant research as they point to the interference of long-term storage in the preservation of molecules that have symbiotic (Liu et al. 2026a) and environmental roles in ecosystems (Emran et al. 2026; Li et al. 2026).

## Data Availability

Data will be available on request.
